# Ocular Manifestation of Granulomatosis with Polyangiitis Presenting as Serous Retinal Detachment: A Case Report

**DOI:** 10.3390/medicina60101690

**Published:** 2024-10-15

**Authors:** Junwoo Lee, Jaehwan Choi, Seung-Young Yu, Kiyoung Kim

**Affiliations:** Department of Ophthalmology, Kyung Hee University Hospital, Kyung Hee University, Seoul 02447, Republic of Korea; jwloph@gmail.com (J.L.);

**Keywords:** granulomatosis with polyangiitis, Wegener’s granulomatosis, serous retinal detachment

## Abstract

*Background*: Ocular involvement is relatively common in granulomatosis with polyangiitis (GPA); however, choroidal involvement is rare. We present a case of serous retinal detachment resulting from choroidal involvement in GPA. *Case presentation*: A 55-year-old male presented to our clinic with bilateral eye redness and pain. Ocular examination revealed bilateral conjunctival injection, and dilation of the episcleral and scleral vessels. Slit-lamp examination revealed anterior chamber cells. Optical coherence tomography (OCT) confirmed serous retinal detachment (SRD) in the left eye. The patient had recently been diagnosed with GPA following a lung biopsy and had received immunosuppressive therapy, including systemic steroids, cyclosporine, mycophenolate mofetil, and rituximab. Five weeks after treatment, the clinical symptoms of the patient, including SRD, improved with alleviation of systemic symptoms. However, tapering systemic steroids and immunosuppressants and discontinuing rituximab led to disease reactivation. OCT demonstrated a recurrence of subretinal fluid, which had previously resolved, and slit-lamp examination showed mild bilateral engorged scleral vessels. *Conclusions*: Choroidal involvement can present as SRD and may indicate disease activity in patients with GPA.

## 1. Introduction

Granulomatosis with polyangiitis (GPA), previously termed Wegener’s granulomatosis, is a rare systemic autoimmune disease characterized by granulomatous inflammation, necrosis, and vasculitis affecting small- to medium-sized blood vessels. Although the etiology remains unknown, GPA commonly involves the upper and lower respiratory tracts and kidneys but can extend to other organs, including the eyes, leading to serious complications if left untreated [1]. Ocular involvement is relatively common, occurring in 30–50% of patients and can manifest in a variety of forms, ranging from episcleritis and scleritis to more severe conditions such as orbital inflammation and, less commonly, retinal and choroidal vasculitis. In some cases, these ocular symptoms may signal disease onset or systemic relapse [2]. Although retinal and choroidal involvement in GPA is less common than anterior segment inflammation, it is significant. Inflammatory changes within the choroid and retina can result in vasculitis, leading to retinal ischemia, macular edema, and subretinal fluid accumulation. Choroidal involvement in GPA can lead to inflammation of the choroidal vessels, resulting in increased vascular permeability and subsequent leakage into the subretinal space. This can cause accumulation of subretinal fluid (SRF) and serous retinal detachment (SRD), often without direct blood–retinal barrier disruption [3,4,5,6]. Previous reports have documented GPA-associated choroidal inflammation leading to complications such as SRF and SRD [7,8,9]. In this report, we present a case of recurrent SRF due to focal choroidal vasculitis in a patient with GPA. Notably, SRF fluctuated with systemic disease activity, resolved with systemic immunosuppressive therapy and recurred during flare-ups. This case highlights the importance of recognizing ocular symptoms as potential indicators of systemic disease activity in GPA and underscores the need for coordinated interdisciplinary management.

## 2. Case Presentation

**Figure 1:**
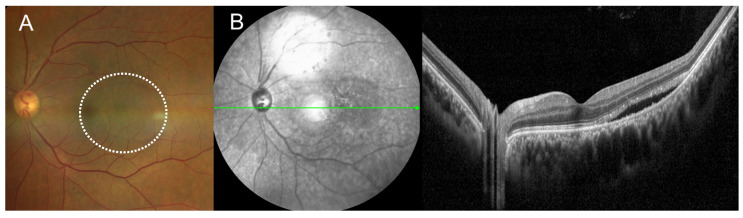
Fundus photograph (**A**) and optical coherence tomography (OCT) images (**B**) at the first visit reveal a small amount of SRF in the temporal side of the macula. (The area of SRF is highlighted with a dashed circle in the fundus photograph).

**Figure 2:**
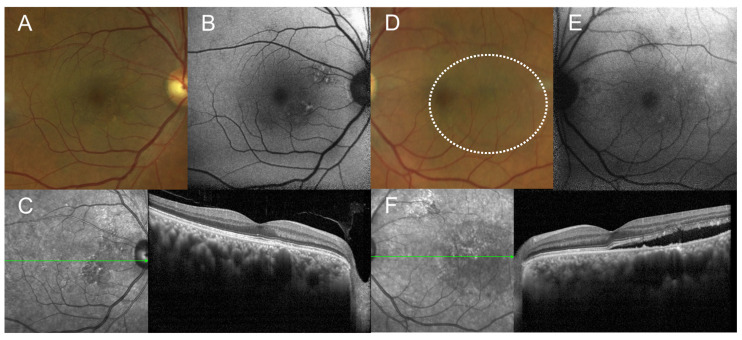
Fundus photographs (**A**,**D**), fundus autofluorescence (**B**,**E**), and OCT images (**C**,**F**) show an increase in SRF in the left eye. In the left eye, the area of SRF is highlighted with a dashed circle on the fundus photograph. (Images (**A**–**C**) correspond to the right eye, while (**D**–**F**) correspond to the left eye).

A 55-year-old male was referred to ophthalmology with bilateral conjunctival injection and eye pain that began three days ago. His best-corrected visual acuity (BCVA) was 20/20 in the right eye and 20/40 in the left eye. Mild conjunctival injection, ocular discomfort, and a small amount of SRF were noted in the left eye (Figure 1). The patient had a recent history of bilateral uveitis, treated one month prior at a local clinic. Systemic evaluation for suspected vasculitis was initiated, with diagnostic results pending. Follow-up appointments were scheduled while awaiting further information. Two weeks later, the patient presented with exacerbated conjunctival injection and ocular pain. His BCVA remained stable at 20/20 in the right eye and 20/40 in the left eye. Slit-lamp biomicroscopy revealed bilateral conjunctival injection with episcleral and scleral vessel dilation. The anterior chamber cell was positive bilaterally. Fundus photography and optical coherence tomography (OCT) confirmed an increase in SRF in the left eye (Figure 2). A comprehensive immunological screening—including complement levels, immunoglobulins, antinuclear antibodies, anti-Sjögren’s syndrome-related antigens (SS-A and SS-B), anti-topoisomerase I (SCL-70), and proteinase 3-antineutrophil cytoplasmic antibodies (PR3-ANCA)—was conducted by the rheumatology department. All immunological markers were within normal limits except for PR3-ANCA, which was positive. Inflammatory markers were notably elevated, with a C-reactive protein (CRP) level of 13.91 mg/dL (reference < 0.5 mg/dL) and an erythrocyte sedimentation rate of 80 mm/h (reference 0–15 mm/h). Chest radiography revealed haziness in the lower right lobe. The patient was treated with systemic corticosteroids and immunosuppressive therapy.

**Figure 3:**
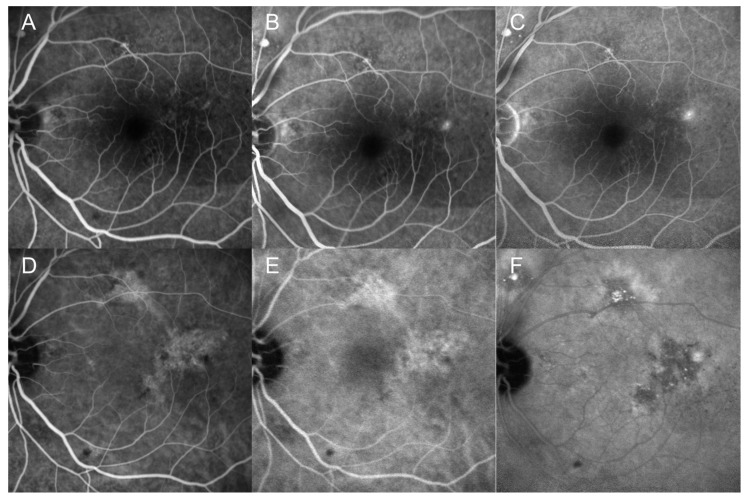
(**A**–**C**) Fluorescein angiography (FA) findings from the first visit. (**A**) Early phase; (**B**) intermediate phase; and (**C**) late phase. FA revealed a significant leakage point. (**D**–**F**) Indocyanine green angiography (ICGA) findings. (**D**) Early phase; (**E**) intermediate phase; and (**F**) late phase. ICGA demonstrated engorged choroidal vessels with focal leakage.

Fluorescein angiography (FA) and indocyanine green angiography (ICGA) were performed. FA revealed early hyperfluorescence with scattered dark spots and mottled hyperfluorescence in the left eye, followed by leakage from the hyperfluorescent spots in the late phase. ICGA demonstrated focal dilation of the choroidal vessels in the affected area, with initial hypercyanescent areas suggestive of choroidal vessel leakage, followed by late hypocyanescence (Figure 3). No evidence of choroidal neovascularization was found. The clinical presentation suggested multiorgan involvement, including suspected ocular, pulmonary, hepatic, renal, and cerebral vascular involvement. Despite ongoing high-dose steroid pulse therapy and cyclophosphamide treatment, the patient continued to experience elevated CRP levels, persistent headaches, and ocular pain. Therefore, the methylprednisolone (MPD) dose was increased from 30 to 60 mg, cyclosporine pulse therapy was escalated from 500 to 1000 mg, and rituximab (500 mg) was administered.

**Figure 4:**
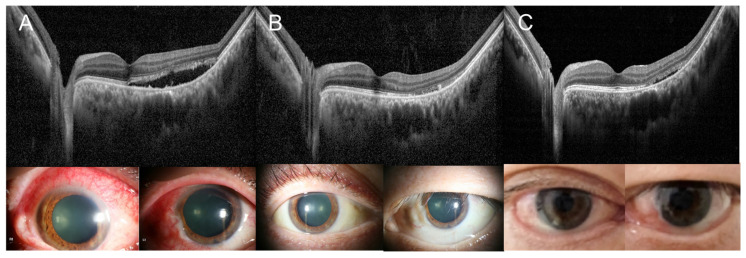
(**A**) Ocular involvement in GPA presenting as scleritis and SRF on OCT. (**B**) Resolution of scleritis with resolved SRF. (**C**) Recurrence of SRF and sign of scleritis.

After five weeks of systemic treatment, the patient demonstrated significant improvement. The conjunctival injection and scleral vessel engorgement resolved. OCT demonstrated resolution of SRF in the left eye. Additionally, the headaches experienced by the patient improved, and laboratory results revealed a decrease in the CRP to 5.91 mg/dL, indicating better disease control. However, during the tapering of steroids and immunosuppressants, and after discontinuing rituximab, the patient experienced ocular pain and mild scleral vessel engorgement in both the eyes again. Blood tests revealed an increase in CRP levels to 8.24 mg/dL, and chest radiography showed increased haziness in the right lower lobe. OCT confirmed the recurrence of SRF in previously resolved lesions (Figure 4). The absence of fever and minimal elevation in procalcitonin levels suggested an exacerbation of vasculitis. Consequently, the re-escalation of cyclophosphamide and steroid doses was considered to manage the GPA.

## 3. Discussion

Ocular manifestations have been documented in approximately 26–50% of patients with GPA [10,11,12], with ocular symptoms serving as the initial indication of the disease in 15.6–19.3% of cases [10,13,14,15,16]. Ocular complications in GPA can be categorized into contiguous and focal forms. The contiguous form involved secondary extension from adjacent paranasal sinuses, characterized by orbital inflammation, proptosis, sinus occlusion, dacryocystitis, extraocular muscle involvement, and optic neuropathy. Conversely, the focal form entails isolated inflammation of the anterior and posterior orbits without sinusitis, attributed to localized necrotizing vasculitis [17]. Common ocular symptoms include proptosis, orbital inflammation, corneal or scleral inflammation, and scleritis [18]. Less commonly, GPA may lead to complications such as rhegmatogenous retinal detachment, SRD, multifocal choroiditis, acute posterior multifocal placoid pigment epitheliopathy, acute retinal necrosis, and neovascular glaucoma [19,20,21].

The mechanism underlying retinal vasculitis in GPA remains unclear, but it is suspected to be associated with ANCA-associated vasculitis. Neutrophils adhere to the vascular endothelium, causing vessel wall injury [22]. Choroidal involvement has been reported in ANCA-associated vasculitis, particularly GPA, with various choroidal manifestations. Kinyoun et al. [23] reported choroidal involvement in GPA due to presumed choriocapillaritis, which led to ischemia and infarction of the choriocapillaris–retinal pigment epithelium–outer neurosensory retina complex. Systemic corticosteroids and immunosuppressive agents were effective in controlling systemic diseases and preserving visual function. Bilateral SRD has also been reported as a form of choroidal involvement [24]. Damato et al. [25] documented SRD secondary to vasculitis and ischemia visible on ICGA. Proia et al. [7] described a case of granulomatous choroiditis in a patient with GPA, emphasizing the role of choroidal vessel inflammation causing SRF accumulation and visual impairment. Lim et al. [8] reported cases of macular retinal vasculitis and choroiditis associated with GPA, resulting in SRD and significant visual disturbances. Takashi et al. [9] described central serous chorioretinopathy (CSC)-like SRD due to choroidal involvement in GPA with fibrin accumulation, noting that reducing steroid doses resolved the SRD.

Unlike previously reported cases, the present case was characterized by localized choroidal vascular inflammation that induced SRD in a patient with GPA. The thickened choroid and the localized leakage points and SRF observed on FA were consistent with those of CSC. Symptom resolution with ongoing treatment, including steroids and immunosuppressants, was observed. While FA excluded retinal vasculitis due to the absence of retinal vascular leakage, ICGA revealed localized dilated choroidal vessels and leakage at the retinal pigment epithelium level, indicating that the SRF was caused by focal choroidal vascular involvement from GPA rather than conventional CSC.

Treatment for GPA typically involves induction therapy with steroids and immunomodulating agents, followed by maintenance therapy with agents, such as cyclophosphamide, glucocorticoids, rituximab, azathioprine, methotrexate, and plasmapheresis, if indicated. In this case, systemic corticosteroid and immunomodulatory therapy resulted in the resolution of systemic symptoms and SRF. The recurrence of SRD during disease reactivation suggests that SRF associated with choroidal vascular involvement may reflect disease activity.

This case highlights the potential for ocular involvement, specifically SRD, associated with disease activity in a patient with GPA. The patient exhibited an improvement in both systemic symptoms and SRD following systemic treatment. However, SRF recurred with disease reactivation during the tapering of steroids and immunosuppressants. Therefore, regular ophthalmic evaluation is essential to prevent visual impairment and facilitate disease management in patients with GPA.

## Data Availability

The data generated in the present study are included in the figures of this article. The data can be obtained from the authors upon reasonable request.
